# Strong One-Dimensional Characteristics of Hole-Carriers in ReS_2_ and ReSe_2_

**DOI:** 10.1038/s41598-019-39540-4

**Published:** 2019-02-25

**Authors:** B. S. Kim, W. S. Kyung, J. D. Denlinger, C. Kim, S. R. Park

**Affiliations:** 1 Department of Physics and Astronomy, Seoul National University, Seoul, 08826 Korea; 2 Center for Correlated Electron Systems, Institute for Basic Science, Seoul, 08826 Korea; 3 Department of Physics, Incheon National University, Incheon, 22012 Korea; 4 Advanced Light Source, Lawrence Berkeley National Laboratory, Berkeley, CA 94720 USA

## Abstract

Each plane of layered ReS_2_ and ReSe_2_ materials has 1D chain structure, from which intriguing properties such as 1D character of the exciton states and linearly polarized photoluminescence originate. However, systematic studies on the 1D character of charge carriers have not been done yet. Here, we report on systematic and comparative studies on the energy-momentum dispersion relationships of layered transition metal dichalcogenides ReS_2_ and ReSe_2_ by angle resolved photoemission. We found that the valence band maximum or the minimum energy for holes is located at the high symmetric Z-point for both materials. However, the out-of-plane (${k}_{z}$) dispersion for ReSe_2_ (20 meV) is found to be much smaller than that of ReS_2_ (150 meV). We observe that the effective mass of the hole carriers along the direction perpendicular to the chain is about 4 times larger than that along the chain direction for both ReS_2_ and ReSe_2_. Remarkably, the experimentally measured hole effective mass is about twice heavier than that from first principles calculation for ReS_2_ although the in-plane anisotropy values from the experiment and calculations are comparable. These observation indicate that bulk ReS_2_ and ReSe_2_ are unique semiconducting transition metal dichalcogenides having strong one-dimensional characters.

## Introduction

Layered transition-metal dichalcogenides (TMDs) have been extensively studied in recent years because of their potential as electronic materials for future devices. Their intriguing characters of the electronic structures such as the valley degeneracy^1^ and spin-valley-layer locking^2–4^, provide added value for valley- and spintronic applications. While some of them even exhibit superconductivity and charge density waves originating from the electron-phonon and electron-electron interactions due to their low electronic dimensionality (e.g. in NbSe_2_^5,6^), most of the studies have been focused on Mo and W compounds due to their semi-conducting properties and close to ideal 2 dimensionality^7–11^.

Very recently, layered ReS_2_ and ReSe_2_ began to draw attention as their layer-layer interactions are much weaker than other layered TMDs^12^. More interestingly, ReS_2_ and ReSe_2_ have one-dimensional (1D) characters in their structure as well as optical and electrical properties due to the formation of Re chain structure stemming from the distortion in the 1T structural phase^12–14^. High-resolution transmission electron microscopy and electron diffraction studies reveal distorted 1T structure with Re chain formation^12^. Such structure distortion is directly related to the high anisotropy in the Raman response from in-plane lattice vibrations^14–19^. It also affects optical properties, e.g., anisotropic character in optical absorption or photoluminescence comes from that of excitons^20–24^. Electrical conductivity is also found to be much higher along the chain direction than the direction perpendicular to the chain^13,25,26^.

As the above-mentioned intriguing 1D properties are determined by the characteristics of hole and electron band dispersions, electronic structure studies by angle resolved photoemission spectroscopy (ARPES) are naturally desired. Especially, the effective mass is a fundamental parameter that can be obtained from the energy and momentum dispersion relationship and governs the electrical and optical properties such as mobility, conductivity, light absorption and photoluminescence. In fact, a couple of ARPES studies on ReS_2_ and ReSe_2_ have been reported and the results indicate an in-plane anisotropy in the hole band dispersion as expected from the chain structures in ReS_2_^27–29^ and in ReSe_2_^30^. However, obtaining very high-quality data needed to determine the effective mass for all directions turned out to be challenging. For example, the photon energy dependence of the valence band dispersion for ReS_2_ appears to be very different among the reported results^27,29^. In addition, direct comparison between measured valence band dispersions of ReS_2_ and ReSe_2_, for which lattice parameters are quite different^31^, are difficult because the data were taken in different Brillouin zones for ReS_2_ and ReSe_2_^29,30^.

In order to resolve aforementioned issues, we have performed systematic ARPES studies on ReS_2_ and ReSe_2_. Our goal is to take data for the entire momentum space which is good enough to do quantitative analysis and obtain in-plane anisotropy in the effective hole mass for the two materials for a comparative study. Our data show a striking difference from what were reported in previous experimental and theoretical studies^29,30,32^. (1) The valence band maximum (VBM) is located at the Z-point for both systems, while it was reported in a previous ARPES study reported that VBM of ReSe_2_ may be located at non-high-symmetric momentum point^30,33^. (2) The ${k}_{z}$ dispersion of ReSe_2_ is much smaller than that of published quasiparticle band structure within the LDA + *GdW* approximation^32^. (3) The effective hole masses along and perpendicular to the chain direction are quite different from the reported experimental and theoretical values^29,30,34^.

## Results and Discussion

### Valence band maximum of ReSe_2_ and ReS_2_

**Figure 1:**
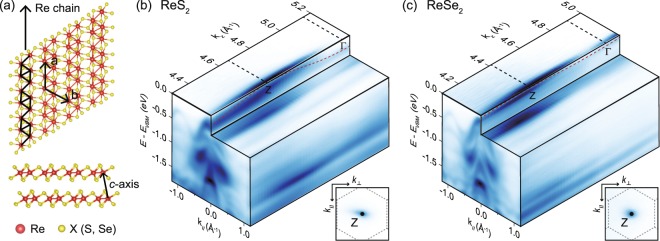
Crystal structure and out-of-plane valence band dispersions. (**a**) Top and side views of the crystal structure of ReX_2_ (X = S, Se). It shows distorted 1 T structure with Re chains indicated by black solid lines. (**b,c**) Intensity plots of ReS_2_ and ReSe_2_ ARPES data in the energy and momentum space, respectively. ***k***_***Z***_ dependent intensities are taken by using different photon energies from 60 eV to 110 eV with 2 eV step. ***k***_***Z***_ is obtained with inner potentials of 17.8 and 12.4 eV for ReS_2_ and ReSe_2_, respectively. ${{\boldsymbol{k}}}_{\parallel }$ and ${{\boldsymbol{k}}}_{\perp }$ are in-plane momentum parallel and perpendicular to the chain, respectively. Red dashed lines are guides to eye for ${{\boldsymbol{k}}}_{{\boldsymbol{z}}}$ dispersion of the top-most valence bands. Insets are 2D constant energy (E = E_VBM_) intensity map in the momentum-space (as functions of ${{\boldsymbol{k}}}_{\parallel }$ and ${{\boldsymbol{k}}}_{\perp }$) at the ${{\boldsymbol{k}}}_{{\boldsymbol{z}}}$ = Z-point. Note that there is a single peak at the zero in-plane momentum point (Z-point).

ReS_2_ and ReSe_2_ are layered materials in which the van der Waals interaction between layers is extremely weak, even weaker than other TMDs^12^. The crystal structure for both ReS_2_ and ReSe_2_ is the so-called distorted 1T structure. Re atoms show a hexagonal network but the structure is distorted to have chain structures as indicated by black lines in Fig. 1(a). The 1D chain structure makes these materials unique among TMDs in that optical and electrical properties carry 1D characteristics^14–26^.

The inner potential can be estimated from the ${k}_{z}$ dispersion of electronic band (Fig. 1(b) and (c)) with the reciprocal lattice vector c*. Based on the results of reported X-ray diffraction measurements^31^, the reciprocal lattice vector c* is calculated to be 1.032 Å^−1^ (0.984 Å^−1^) for ReS_2_ (ReSe_2_). The inner potential is estimated to be *V*_0_ = 17.8 and 12.4 eV for ReS_2_ and ReSe_2_. These estimated values are similar to those of other TMDs^35,36^.

ARPES experiments are performed on ReS_2_ and ReSe_2_ to obtain the energy-and-momentum dispersion of the hole carriers. ARPES intensities as a function of the energy referenced to the valence band maximum (E_VBM_) are mapped along two momentum directions, parallel to chain (${k}_{\parallel }$) and perpendicular to the layer (${k}_{z}$) (Fig. 1(b,c)). While several band dispersions are observed within the energy range, the top-most valence band is of interest as it determines the low energy properties of the materials such as electrical conductivity. Due to the layered structure, the top-most bands of ReS_2_ and ReSe_2_ show relatively weak dispersions along ${k}_{z}$ than along in-plane momentum. Interestingly, we observed as shown in Fig. 1 that the ${k}_{z}$ dispersion of ReSe_2_ (about 20 meV) is even weaker compared to that of ReS_2_ (about 150 meV) which is known as a material with very weak inter-layer interaction^12^. Therefore, our results show an evidence for even smaller interaction between layers in ReSe_2_.

Our photon energy dependence data reveal that VBM is located at Z for both ReS_2_ and ReSe_2_ as indicated by the red dashed lines in Fig. 1(b,c). While previous ARPES studies also showed that VBM of ReS_2_ is located at Z, VBM of ReSe_2_ has been under debate. Hart *et al*. reported that the ${k}_{z}$ for VBM of ReSe_2_ is the same as the Z-point but the in-plane momentum was reported to be non-zero^30^. More recently, Eickholt *et al*. reported two VBM of ReSe_2_^33^. One of them is at Z and the other is away from Z. But their experiment could not decide which is global VBM, since data quality is not good enough. The global VBM of ReSe_2_ can be decided to be located at Z due to high quality data. Please refer to the supplementary materials for more details. In fact, we find that ReSe_2_ result about VBM is consistent with a recent theoretical prediction as well^32^.

### Directional dependence of effective hole masses in ReS_2_ and ReSe_2_

**Figure 2:**
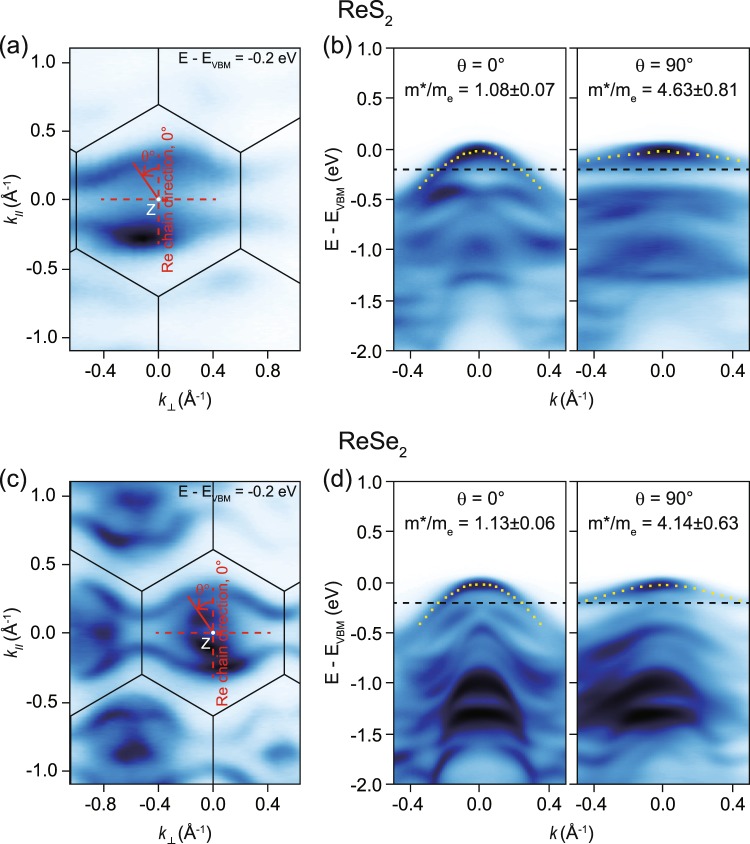
In-plane valence band dispersions near the valence band maximum. Constant energy (E-E_VBM_ = − 0.2 eV) ARPES maps of ReS_2_ (**a**) and ReSe_2_ (**c**). The photon energy used for the experiment was 70 eV for ReS_2_ and 68 eV for ReSe_2_. For these photon energies, ${{\boldsymbol{k}}}_{{\boldsymbol{z}}}$ = Z point (white dot) where VBM is located is included in the data. Red solid and dashed lines in (**a,c**) indicate the direction parallel and perpendicular to the Re chain direction, respectively. θ is defined as the relative angle from the direction along the chain as shown in the figure. The honey comb structured line indicates the projected Brillouin zone boundary. (**b,d**) High symmetric cuts along (θ = 0°) and perpendicular (θ = 90°) to the Re chain. The dotted lines in (**b,d**) are quadratic fit to the top-most bands. The effective masses of hole carriers of ReX_2_ extracted from the fitting functions are shown in the figures.

In order to investigate the effective mass of the hole carrier, we analyze ARPES data obtained in the in-plane momentum space that includes the Z-point. As shown in Fig. 2(a,c), constant energy maps of ARPES intensities of ReS_2_ and ReSe_2_ at E-E_VBM_ = − 0.2 eV show two-fold symmetry and strong anisotropic band contours which are not closed along the direction perpendicular to the chain. These observations indicate much smaller band dispersion along the direction perpendicular to the chain. The top-most band dispersions, which we are interested in, along the chain are much stronger than those along the other for both ReS_2_ and ReSe_2_. For quantitative analysis, we try to fit the band dispersions with a quadratic function for which the effective mass is a free parameter^32^. The dotted lines in Fig. 2(b,d) indicate the fit functions. So, obtained effective masses along the direction perpendicular to the chain (4.63 m_e_ for ReS_2_ and 4.14 m_e_ for ReSe_2_) are much heavier than the effective masses along chain (1.08 m_e_ for ReS_2_ and 1.13 m_e_ for ReSe_2_). That is, the effective mass along the chain is about 4 times lighter than that perpendicular to the chain for both ReS_2_ and ReSe_2_. This in-plane anisotropy value in the effective hole mass is the largest among semiconducting TMDs^37,38^.

**Figure 3:**
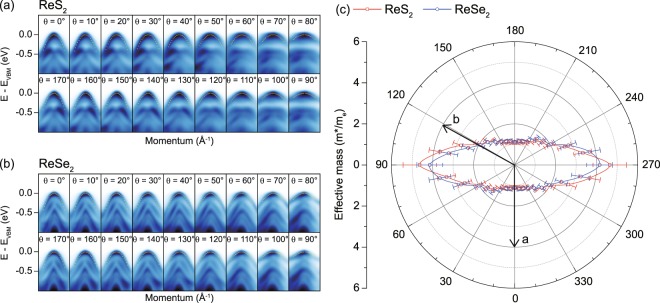
Anisotropic effective hole masses. ARPES cut data along the in-plane momentum defined by the angle θ (see Fig. 2(a) for definition) for (**a**) ReS_2_ and (**b**) ReSe_2_. All the data sets are centered around the Z-point at which VBM is located. The dotted lines indicate a quadratic function fitting the dispersions of the top-most bands. The effective mass from the quadratic fit function for each angle can be extracted and is plotted for ReS_2_ and ReSe_2_ in (**c**). Arrows indicate crystallographic orientation of ReX_2_.

The valence band dispersion can also be analyzed for different theta angle and corresponding effective hole mass can be obtained. Shown in Fig. 3(a,b) are ARPES data along in-plane momentum set by the θ angle defined in Fig. 2(a). The data are subsequently analyzed and the corresponding effective hole mass is obtained for a systematic study of direction dependence. We notice the top-most band can be fitted well with a quadratic function indicated by dotted lines, which makes us confident in our analysis. The extracted effective mass from the quadratic function is plotted in polar coordinate as a function of the theta angle in Fig. 3(c). The plot clearly shows two-fold symmetry and strong in-plane anisotropy of the effective hole mass for both ReS_2_ (red) and ReSe_2_ (blue). There is an important point to discuss in comparison with the results of first principles calculations on ReS_2_. The experimentally observed effective mass is about twice larger than that from the first principles calculations. The effective mass from the first principles calculations is 2.4 m_e_ along the direction perpendicular to the chain and 0.8 m_e_ along the chain^27^. The electron-electron and electron-phonon interactions or atomic spin-orbit coupling of Re atom which were not considered in the calculation may play a crucial role in the clear enhancement of the effective hole mass.

## Conclusions

In this study, we performed systematic ARPES studies of ReS_2_ and ReSe_2_ to reveal the energy-momentum dispersion relationships of the top most valence bands. We found that ReSe_2_ have much smaller ${k}_{z}\,$dispersion than ReS_2_, indicating the more 2D-like feature in ReSe_2_ than in ReS_2_. We systematically investigated in-plane directional dependence of the effective hole masses of ReS_2_ and ReSe_2_. The effective masses show strong anisotropy, about 4 times lighter along the chain than the direction perpendicular to the chain. In-plane anisotropy of the hole effective masses in ReS_2_ and ReSe_2_ is larger than that in black phosphorus which also shows anisotropic electrical and optical properties^39,40^. Therefore, ReS_2_ and ReSe_2_ are quasi 1D materials in terms of the low energy hole carrier dynamics, which makes ReS_2_ and ReSe_2_ promising bulk materials for 1D semiconducting electronics. The effective mass of ReS_2_ observed by ARPES is found to be significantly enhanced compared to that from first principles calculations. Electron-electron and electron-phonon interactions or atomic spin-orbit coupling of Re atom may be attributed to the mass enhancement^41^. The quasi 1D character of the hole carriers as well as possibility of the electron-electron and electron-phonon interactions may lead to charge density wave order if enough amount of hole carriers are doped into ReS_2_ and ReSe_2_^42^.

## Methods

### ARPES measurement

We performed ARPES experiments at the beamline 4.0.3.2 (MERLIN) of the Advanced Light Source at the Lawrence Berkeley National Laboratory equipped with VG-Scienta R8000 electron analyzer. All samples are cleaved *in-situ* and data were taken at 200 K to avoid the charging effect in a vacuum better than 6 × 10^−11^ Torr. with linearly polarized light. For the k_z_ dependence experiment, photon energies between 60 and 110 eV with 2 eV energy step were used. The total energy resolution was better than 20 meV with a momentum resolution of 0.004 Å^−1^.

## Supplementary information

Supplementary Information
