# Supplementary material for: Strong One-Dimensional Characteristics of Hole-Carriers in ReS2 and ReSe2
Source: Sci Rep. 2019 Feb 25;9:2730. doi: 10.1038/s41598-019-39540-4 (PMC6389895; doi:10.1038/s41598-019-39540-4)
Supplement: Supplementary file 1 — Supplementary Information [file 41598_2019_39540_MOESM1_ESM.pdf]

Supplementary Materials: Strong One-Dimensional Characteristics of Hole-Carriers in ReS_2 and $ReSe_2$

Beom Seo Kim,[1,2,3] Wonshik Kyung,[1,2,4] Jonathan D. Denlinger,[4] Changyoung Kim,[1,2,*] and Seung Ryong Park[3,†]

[1]*Department of Physics and Astronomy, Seoul National University (SNU), Seoul 08826, Republic of Korea*
[2]*Center for Correlated Electron Systems, Institute for Basic Science (IBS), Seoul 08826, Republic of Korea*
[3]*Department of Physics, Incheon National University, Incheon 406-772, Korea*
[4]*Advanced Light Source, Lawrence Berkeley National Laboratory, Berkeley, California 94720, USA*
(Dated: December 14, 2018)

PACS numbers:

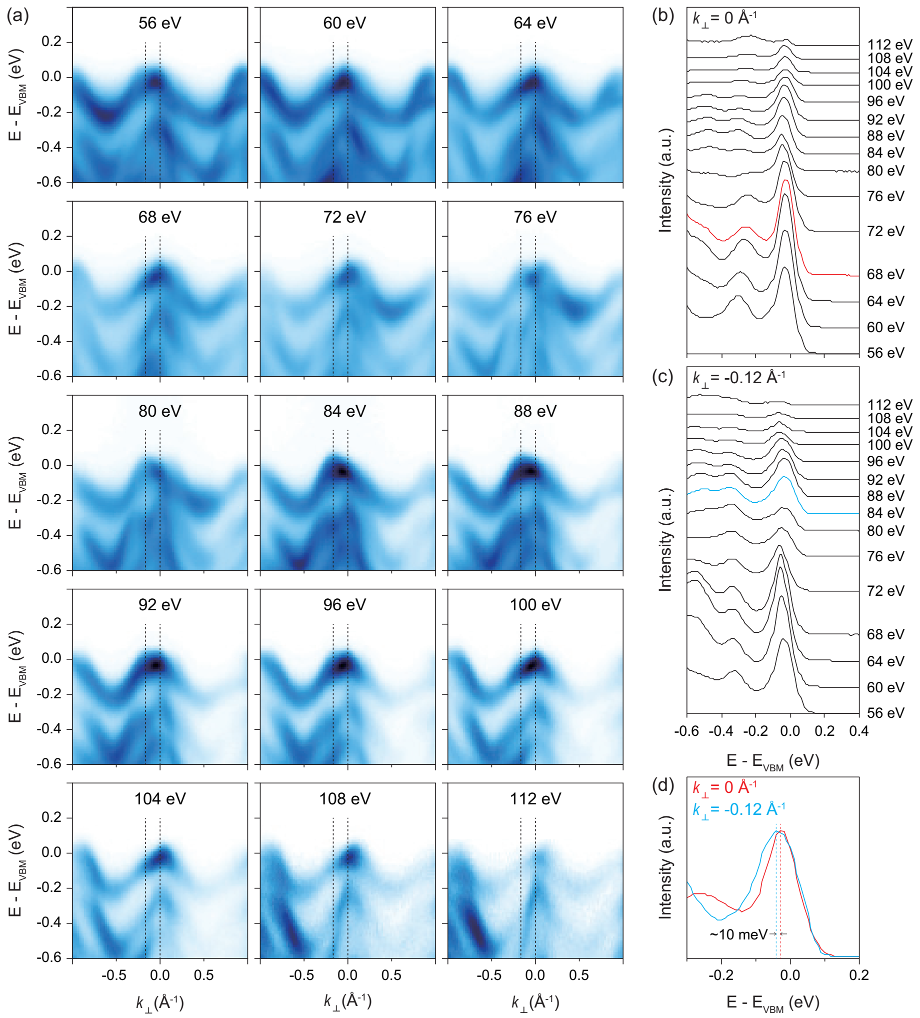

FIG. S1: (a) High symmetry cuts perpendicular to the Re chain for various photon energies from 56 eV to 112 eV. Energy step is 4 eV. Black dotted lines indicate positions of the in-plane momentum $k_\perp = 0$ Å^{-1} and $k_\perp = -0.12$ Å^{-1}. Energy distribution curves (EDCs) of (b) $k_\perp = 0$ Å^{-1} and (c) $k_\perp = -0.12$ Å^{-1} for different photon energies. Red and blue lines are VBM of $k_\perp = 0$ Å^{-1} and $k_\perp = -0.12$ Å^{-1}, respectively. (d) Expanded view of the two EDCs (red and blue curves in (b) and (c), respectively) for a better comparison.

---

*Electronic address: changyoung@snu.ac.kr
†Electronic address: AbePark@inu.ac.kr

We address the issue on the position of the global valence band maximum (VBM) in $ReSe_2$ in this supplementary materials. A very recent experimental report discusses the position of the global VBM for $ReSe_2$[1]. It was found that the VBM is located at one of the two momenta. The first one is the Z-point at which the in-plane momentum is zero. The other is a non-high symmetry point at which $k_{\|}$ is zero and $k_{\perp}$ is -0.12 Å^{-1}. However, which one of the two could not be determined due to the limit of experimental resolution. Our data clearly show that the global VBM is located at the Z-point. We find that the binding energy of the top most band is about 10 meV higher at the Z-point ($k_z = 4.43$ Å^{-1}), which can be taken by 68 eV photon energy, than any other point (Fig. S1 (d)).

[S1] P. Eickholt, J. Noky, E. F. Schwier, K. Shimada, K. Miyamoto, T. Okuda, C. Datzer, M. Drüppel, P. Krüger, M. Rohlfing, and M. Donath, Phys. Rev. B **97**, 165130 (2018).
